# Educational Attainment and US Drug Overdose Deaths

**DOI:** 10.1001/jamahealthforum.2023.3274

**Published:** 2023-10-06

**Authors:** David Powell

**Affiliations:** 1 RAND, Arlington, Virginia

## Abstract

**Importance:**

Educational attainment in the US is associated with life expectancy. As the opioid crisis worsens, it is critical to understand how overdose death rate trends evolve across education groups.

**Objective:**

To investigate the association between educational attainment and overdose death rates, with emphasis on trends during the COVID-19 pandemic.

**Design, Setting, and Participants:**

This cross-sectional study used National Vital Statistics System Mortality Multiple Cause-of-Death data describing overdose death rates in the US by educational attainment from January 1, 2000, to December 31, 2021, with a focus on 2018 to 2021. Overdose deaths were aggregated by year and educational level for decedents aged 25 years or older.

**Exposure:**

Educational attainment, categorized as no high school (HS) diploma, HS diploma (or General Educational Development) but no college, some college but no bachelor’s degree, and bachelor’s degree or more.

**Main Outcomes and Measures:**

The main outcomes were rates of all overdose deaths, overdose deaths involving opioids, and overdose deaths involving synthetic opioids.

**Results:**

Of 912 057 overdose deaths with education information from 2000 to 2021 (mean [SD] age at death, 44.9 [12.3] years; 64.1% male), there were 625 400 deaths (68.6%) among individuals with no college education and 286 657 deaths (31.4%) among those with at least some college. The overdose death rate was 19.9 per 100 000 population. From 2018 to 2021, there were 301 557 overdose deaths, including 58 319 (19.3%) among individuals without an HS diploma, 153 603 (50.9%) among people with an HS diploma, 64 682 (21.4%) among individuals with some college, and 24 953 (8.3%) among individuals with a bachelor’s degree. There were 3324 overdose deaths (1.1%) among American Indian or Alaska Native individuals, 2968 (1.0%) among Asian American or Pacific Islander individuals, 49 152 (16.3%) among Black individuals, 31 703 (10.5%) among Hispanic individuals, 211 359 (70.1%) among White individuals, and 3051 (1.0%) among multiracial individuals. From 2018 to 2021, the overdose death rate was 33.4 per 100 000 population, the opioid-related overdose death rate was 24.2 per 100 000 population, and the synthetic opioid overdose death rate was 19.1 per 100 000 population. From 2018 to 2021, the overdose death rate for those without a HS diploma increased by 35.4 per 100 000 population compared with 1.5 per 100 000 population for those with a bachelor’s degree. This differential growth was primarily due to increased rates of death involving synthetic opioids.

**Conclusions and Relevance:**

In this cross-sectional study, lower educational attainment was found to be associated with higher growth in overdose deaths. As the opioid crisis has transitioned to fentanyl and polysubstance use, overdose deaths have become more prevalent in groups with lower socioeconomic status, potentially exacerbating existing life-expectancy disparities.

## Key Points

**Question:**

Was educational attainment associated with overdose death rate growth in the US from 2000 to 2021?

**Findings:**

In this cross-sectional study of 912 057 overdose deaths in the US from 2000 to 2021, overdose deaths increased sharply among individuals without any college education. The overdose death rate increased substantially between 2018 and 2021 for those without a high school diploma, primarily due to increases in deaths with synthetic opioid involvement.

**Meaning:**

In this study, educational attainment, an important component of socioeconomic status, was found to be associated with overdose deaths, especially during the COVID-19 pandemic.

## Teaser

This cross-sectional study uses data from the National Vital Statistics System to examine changes in rates of overdose deaths by level of educational attainment in the US from 2000 to 2021.

## Introduction

Drug overdose mortality continues to surge in the US,^1^ contributing to declining life expectancy.^2^ The literature has dedicated special attention to the demographics of the crisis, documenting overdose death rates by sex, race and ethnicity, and age.^1,3,4,5,6,7,8,9,10,11,12,13,14,15^ There is less research on disparities by educational attainment, especially during the COVID-19 pandemic and despite its central importance in broader discussions of deaths of despair.^16,17,18,19^ Overdose deaths were previously found to be important contributors to the education gradient in mortality from 1992 to 2011^20^ and in the deaths of despair literature through 2013.^18^ More recent work has studied overdose death rates by educational attainment through 2019 or earlier,^21,22,23^ finding that differences in overdose rates by level of education have continued to grow. Examining the trajectory of disparities in overdose deaths for recent years is critical for understanding the evolving relationship between socioeconomic status and life expectancy.^24^ More broadly, the education gradient in mortality and life expectancy has been of central interest to many fields.^20,25,26,27,28,29,30,31,32,33,34,35^ As the opioid crisis shifts into a different phase dominated by polysubstance use,^36,37^ understanding disparities by educational attainment gains importance. There is little information about the role of the opioid crisis in affecting mortality rates by educational level during the COVID-19 pandemic.

Recent waves of the opioid crisis have disproportionately impacted populations that were less affected by the first wave of the crisis, evidenced by the recent disparate rise in mortality in the Black population.^7,36,37^ The COVID-19 pandemic further exacerbated the overdose crisis, with especially large overdose death rate growth among Black and Hispanic individuals.^5,38,39^ Less research has documented how overdose death rates vary by educational level in the recent waves of the crisis and during the pandemic. Understanding who is most impacted by overdose deaths provides critical information about how resources, such as access to treatment and naloxone,^40,41,42^ should be more effectively allocated while offering evidence of the factors associated with the rising incidence of overdose rates^43,44,45^ and the importance of socioeconomic stressors.^46,47^

Educational level is a critical determinant of socioeconomic status and is associated with a host of factors, such as income, employment, marital status, access to health care, and housing security, that may be independently associated with overdose propensities.^47,48,49^ Educational level–specific overdose rates provide additional context regarding disparities by race and ethnicity and by sex given educational differences across demographic groups. This study assessed overdose death rates by educational attainment using data from more recent waves of the opioid crisis and during the COVID-19 pandemic while examining educational gradients by sex and across racial and ethnic groups.

## Methods

### Data Source

In this cross-sectional study, I used National Vital Statistics System (NVSS) Mortality Multiple Cause-of-Death data,^50^ the census of deaths in the US from January 1, 2000, to December 31, 2021. I coded deaths as overdoses using the *International Statistical Classification of Diseases and Related Health Problems, Tenth Revision* external cause of injury codes X40-X44, X60-64, X85, and Y10-Y14. Much of the analysis studied all overdose deaths given that opioid involvement is not always correctly specified^51^ and rates of unclassified overdose deaths are potentially associated with educational level.^52^ I limited the study to overdose deaths of US residents in the 50 states and the District of Columbia. The study used public deidentified data, and the RAND institutional review board determined that the study was exempt from review, with a waiver of informed consent, because it did not involve human participants. I followed the Strengthening the Reporting of Observational Studies in Epidemiology (STROBE) reporting guideline for cross-sectional studies.

To quantify opioid involvement, I used *International Statistical Classification of Diseases and Related Health Problems, Tenth Revision (ICD-10)* multiple-cause-of-death codes T40.0 (opium), T40.1 (heroin), T40.2 (natural and semisynthetic opioids excluding heroin), T40.3 (methadone), T40.4 (synthetic opioids excluding methadone), and T40.6 (other and unspecified narcotics). Demographic information is generally determined by funeral directors, attending physicians, medical examiners, and coroners. They often rely on an informant, typically a close family member.^53^ The 2018 data were the first in which educational level was reported in the NVSS using the 2003 revision of the death certificate for all deaths (unless missing entirely). In prior years, some deaths still used the 1989 revision categorizations. The consistency from 2018 to 2021 permitted the construction of more granular education categories. For the 2018 to 2021 period, I studied 4 nonoverlapping education categories: (1) no high school (HS) diploma, (2) HS diploma (or General Educational Development) but no college, (3) some college but no bachelor’s degree, and (4) a bachelor’s degree or more. The analysis included decedents aged 25 years or older. Deaths for which education information was missing were excluded.

I constructed 2 education categories for 2000 to 2021. For both the 1989 and 2003 death certificate revisions, it is reported whether the decedent attended any college, so I aggregated deaths into no college and at least some college.

I used the following race categories for 2018 to 2021: American Indian or Alaska Native, Asian American or Pacific Islander, Black, White, and multiracial. The Asian American or Pacific Islander and multiracial populations were aggregated from more specific categories. While aggregating obscured heterogeneity within each group, these aggregations are standard in the literature given that the sample sizes are otherwise small (eg, multiracial was reported in 1.0% of overdose deaths and Asian American or Pacific Islander was reported in 1.0%). The NVSS also provides information on Hispanic ethnicity. Combining this information, this study used the following nonoverlapping race and ethnicity categories: non-Hispanic American Indian or Alaska Native, non-Hispanic Asian American or Pacific Islander, non-Hispanic Black, Hispanic, non-Hispanic White, and non-Hispanic multiracial.

I calculated population totals by year, education category, and race and ethnicity using the 2000-2021 American Community Survey (ACS) for individuals aged 25 years or older.^54^ Beginning in 2020, the US Census Bureau updated how it collected and processed race and ethnicity data, which affected reported population sizes.^55^ The final variables are expressed as per 100 000 population.

### Statistical Analysis

This repeated cross-sectional study analyzed trends in overdose deaths from 2000 to 2021, stratified by educational attainment, with a focus on the 2018 to 2021 period. For the 2018 to 2021 period, I studied all overdose deaths, overdose deaths involving opiates, and overdose deaths involving synthetic opioids. I also studied stratifications based on race and ethnicity and by sex. This descriptive study did not involve statistical tests. I conducted the analyses using Stata, version 17.0 (StataCorp LLC).

## Results

Education information was missing for 3.2% of overdose deaths from 2018 to 2021 and for 4.0% of overdose deaths from 2000 to 2021. A total of 912 057 overdose deaths among individuals aged 25 years or older from 2000 to 2021 had education information (mean [SD] age at death, 44.9 [12.3] years; 35.9% female and 64.1% male). There were 625 400 deaths (68.6%) among people with no college experience and 286 657 deaths (31.4%) among those with at least some college. The overdose death rate during this period was 19.9 per 100 000 population. From 2018 to 2021, there were 301 557 overdose deaths among individuals aged 25 years or older with education information, including 58 319 (19.3%) among people without an HS diploma, 153 603 (50.9%) among those with an HS diploma, 64 682 (21.4%) among those with some college experience, and 24 953 (8.3%) among those with a bachelor’s degree. Of these deaths, 3324 (1.1%) were among American Indian or Alaska Native individuals, 2968 (1.0%) among Asian American and Pacific Islander individuals, 49 152 (16.3%) among Black individuals, 31 703 (10.5%) among Hispanic individuals, 211 359 (70.1%) among White individuals, and 3051 (1.0%) among multiracial individuals. The overdose death rate during this period was 33.4 per 100 000 population, the opioid-related overdose death rate was 24.2 per 100 000 population, and the synthetic opioid overdose death rate was 19.1 per 100 000 population.

**Figure 1.**
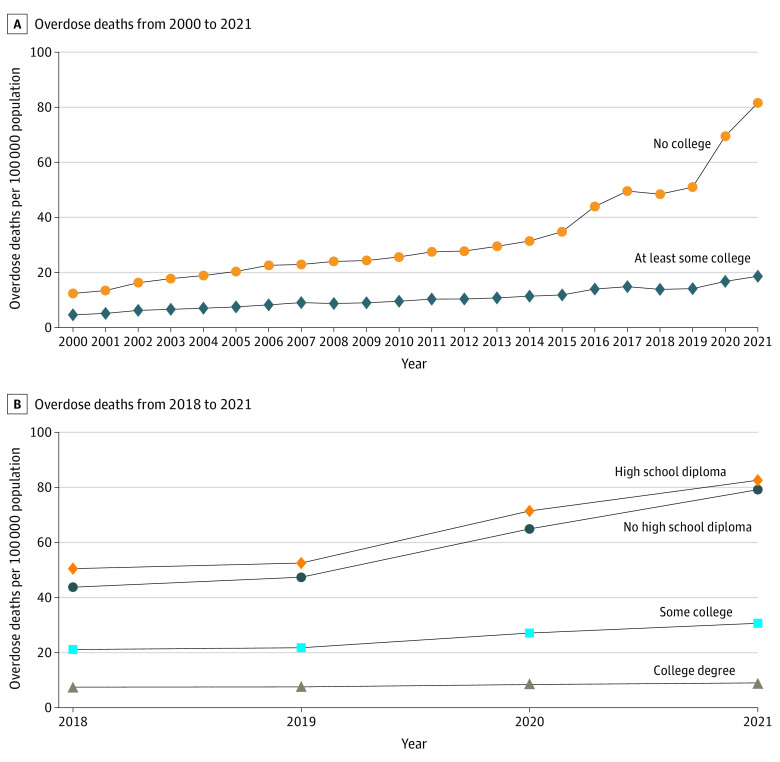
Trends in Overdose Deaths per 100 000 Population by Educational Attainment Analysis based on National Vital Statistics System Mortality Multiple Cause-of-Death data. Overdose deaths were defined as *International Statistical Classification of Diseases and Related Health Problems, Tenth Revision* external cause of injury codes X40-X44, X60-64, X85, or Y10-Y14. Educational attainment stratification used information reported on death certificates.

Overdose death rates increased from 2000 to 2021 for both education categories, but the growth rates differed considerably (Figure 1A). In almost all years, the group without any college education experienced faster growth. For the population without any college education, the overdose death rate was 12.4 per 100 000 population in 2000 and rose to 81.6 per 100 000 population in 2021. For the population with at least some college, the 2000 overdose death rate was 4.6 per 100 000 population, growing to 18.6 per 100 000 population in 2021. There was especially fast growth in overdose death rates in 2016 and 2017 among the population without any college education, followed by a plateau in 2018 and 2019. However, in 2020, the overdose death rate in this group increased by an additional 18.6 per 100 000 population, followed by an increase of 12.1 per 100 000 population in 2021. The group with at least some college education experienced a slower increase in overdose death rate of 2.6 per 100 000 population in 2020 and 1.9 per 100 000 population in 2021. The overdose death rate for the population with no college increased by 38.6 per 100 000 population from 2000 to 2019. This was followed by an increase of 30.6 per 100 000 population from 2019 to 2021, the largest 2-year increase since at least 2000. In comparison, the overdose death rate for the group with at least some college increased by 9.5 per 100 000 population from 2000 to 2019, followed by an increase of 4.5 per 100 000 population from 2019 to 2021.

Studying the 2018 to 2021 period and more granular education groups, individuals in the lowest educational attainment category (no HS diploma) experienced an increase in overdose deaths of 35.4 per 100 000 population, compared with 32.1 per 100 000 population for those with an HS diploma, 9.5 per 100 000 population for those with some college, and 1.5 per 100 000 population for those with a bachelor’s degree (Figure 1B). There were substantial differences across education categories in 2018, and the heterogeneous increase in overdose death rates generally exacerbated those disparities. The population with a bachelor’s degree experienced 9.0 overdose deaths per 100 000 population in 2021, while the population with an HS diploma experienced 82.6 per 100 000 population. The population without an HS diploma had a similar overdose death rate as the group with an HS diploma in 2021, with 79.2 per 100 000 population, whereas the population with some college experience had 30.7 per 100 000 population. These differences were similar when focusing on overdose deaths reporting opioid involvement (eFigure 1 in Supplement 1) and when using age-adjusted overdose death rates (eFigure 2 in Supplement 1).

**Figure 2.**
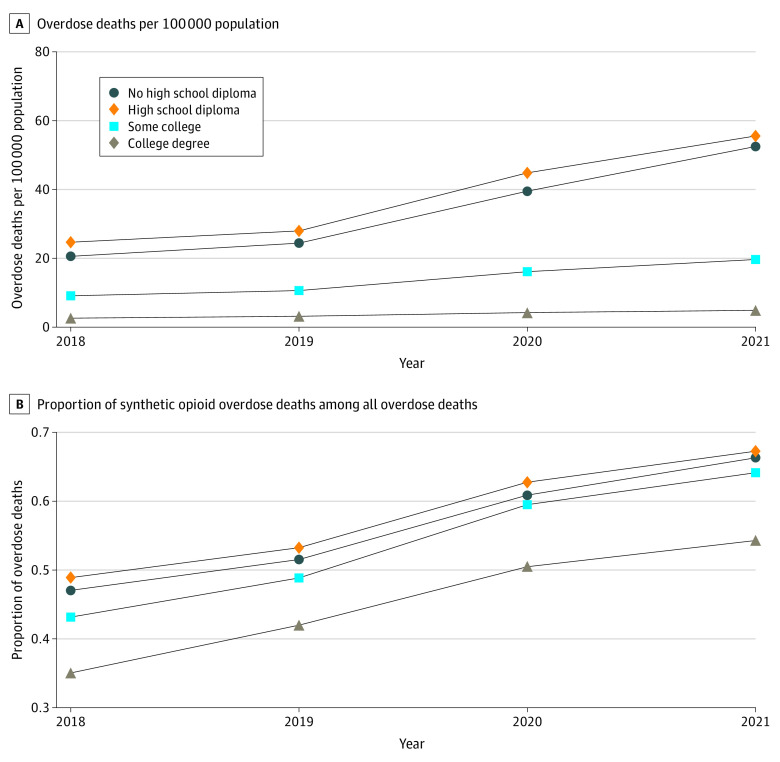
Trends in Synthetic Opioid Overdose Deaths by Educational Attainment, 2018-2021 Analysis based on National Vital Statistics System Mortality Multiple Cause-of-Death data. Overdose deaths with synthetic opioid involvement were defined as *International Statistical Classification of Diseases and Related Health Problems, Tenth Revision* multiple-cause-of-death code T40.4 (synthetic opioids excluding methadone). Educational attainment stratification used information reported on death certificates. B, Share of overdose deaths (between 0 and 1) with reported T40.4 involvement.

Much of this differential growth was due to overdose deaths involving synthetic opioids (Figure 2A). From 2018 to 2021, the synthetic opioid overdose death rate increased by 31.9 per 100 000 population for individuals without an HS diploma, 30.9 per 100 000 population for those with an HS diploma, 10.5 per 100 000 population for those with some college, and 2.3 per 100 000 population for those with a bachelor’s degree. The share of overdose deaths involving synthetic opioids increased for all groups at about the same rate (Figure 2B).

**Figure 3.**
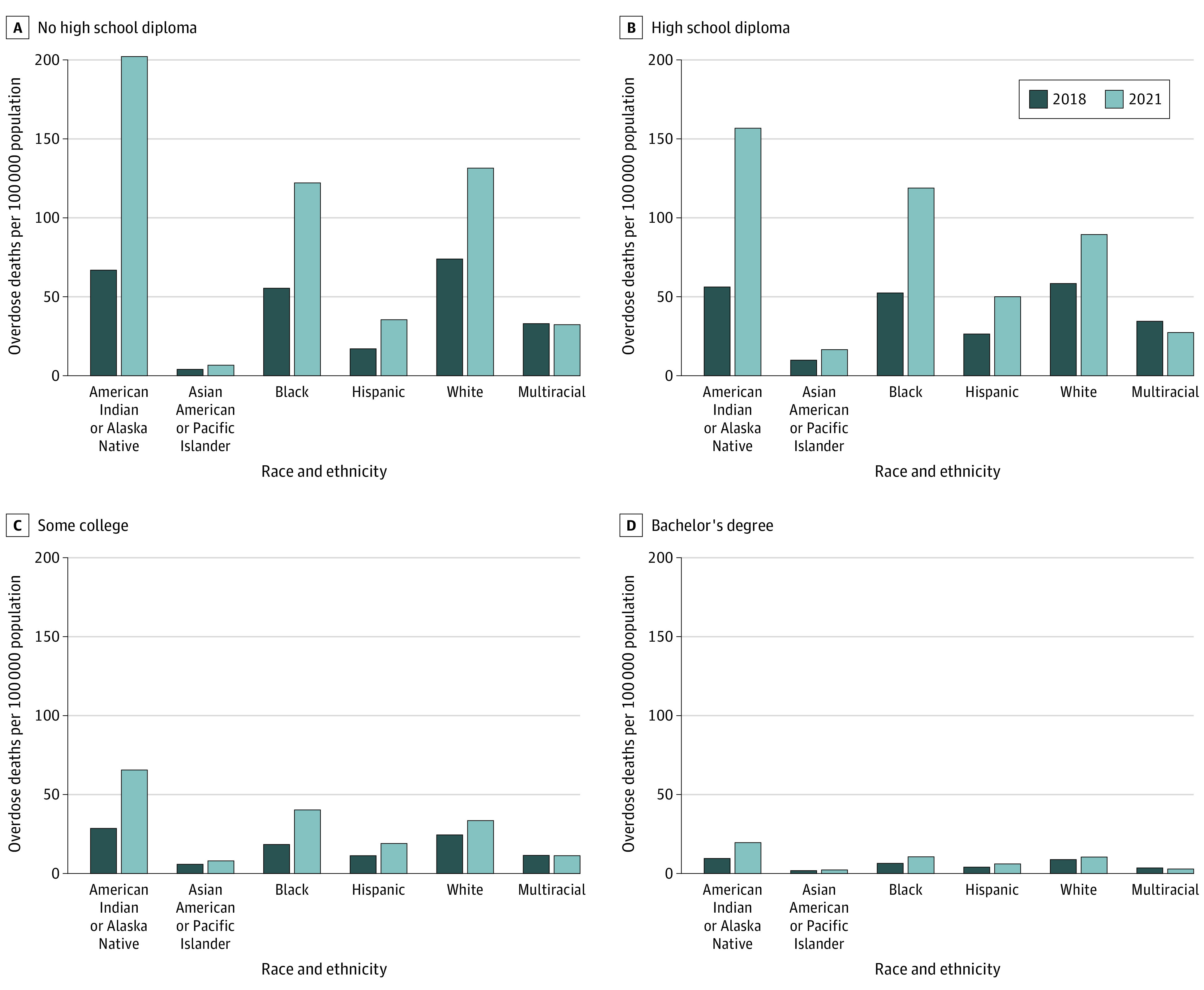
Overdose Deaths per 100 000 Population by Race and Ethnicity in 2018 and 2021 Analysis based on National Vital Statistics System Mortality Multiple Cause-of-Death data. Overdose deaths were defined as *International Statistical Classification of Diseases and Related Health Problems, Tenth Revision* external cause of injury codes X40-X44, X60-64, X85, or Y10-Y14. Educational attainment stratification used information reported on death certificates. All listed race categories implicitly refer to the non-Hispanic population.

Among individuals without an HS diploma, large differences were observed both in educational attainment levels and in growth of overdose death rates. American Indian or Alaska Native, Black, and White individuals without an HS diploma had substantially higher death rates compared with the rest of the population (Figure 3A). In 2021, the American Indian and Alaska Native population without an HS diploma experienced 202 overdose deaths per 100 000 population, an increase of 135 per 100 000 population from 2018. Among those without an HS diploma, White individuals continued to have higher overdose death rates (132 per 100 000 population in 2021) than Black individuals (122 per 100 000 population in 2021), although Black individuals experienced faster growth in overdose death rates from 2018 to 2021. In 2021, Hispanic individuals without an HS diploma had 35.7 overdose deaths per 100 000 population compared with 17.0 per 100 000 population in 2018. Asian American or Pacific Islander individuals had 6.9 deaths per 100 000 population, and multiracial individuals had 32.4 per 100 000 population in 2021.

In the population with an HS diploma, the American Indian and Alaska Native population had the highest overdose death rate and the highest rate of growth in overdose deaths from 2018 to 2021 (Figure 3B). Black individuals had the second highest rate of growth in overdose deaths and, in 2021, had 119 deaths per 100 000 population. White individuals with an HS diploma had 89 per 100 000 population in 2021.

Overdose death rates were smaller at higher educational levels (Figure 3C). For individuals with some college education, the American Indian and Alaska Native population experienced an additional 37.1 per 100 000 population in 2021 compared with 2018, while Black individuals experienced an additional 21.9 per 100 000 population.

Among individuals with a bachelor’s degree, American Indian or Alaska Native individuals had the highest overdose death rate and experienced an additional 10.1 per 100 000 population in 2021 compared with 2018. Black and White individuals in this group had similar overdose death rates in 2021 (10.7 and 10.4 per 100 000 population, respectively). eFigure 3 in Supplement 1 provides the same rates as Figure 3 but grouped by race and ethnicity.

**Figure 4.**
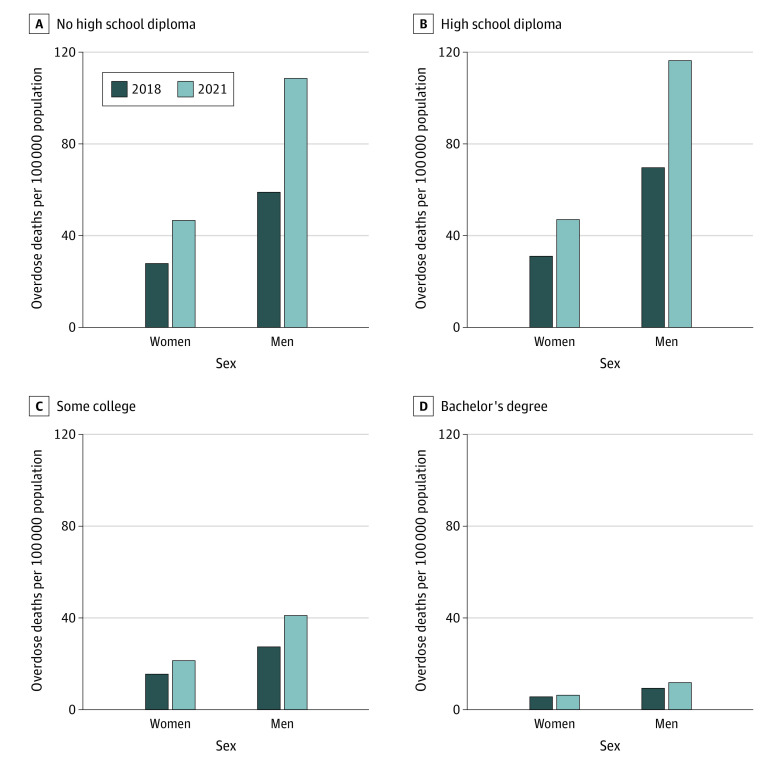
Overdose Deaths per 100 000 Population by Sex and Educational Attainment in 2018 and 2021 Analysis based on National Vital Statistics System Mortality Multiple Cause-of-Death data. Overdose deaths were defined as *International Statistical Classification of Diseases and Related Health Problems, Tenth Revision* external cause of injury codes X40-X44, X60-64, X85, or Y10-Y14. Education attainment stratification used information reported on death certificates.

Next, I studied overdose disparities by sex and educational attainment (Figure 4). Men had higher overdose death rates than women for all education groups. They also experienced faster growth at every educational level. For those without an HS diploma, the overdose death rate among men increased by 49.6 per 100 000 population compared with 18.8 per 100 000 population among women from 2018 to 2021. This growth decreased monotonically by educational level for both men and women: women with a bachelor’s degree experienced an additional 0.8 overdose deaths per 100 000 population in 2021 compared with 2018, while men with a bachelor’s degree experienced an additional 2.4 per 100 000 population.

Men without an HS diploma had 59.0 overdose deaths per 100 000 population in 2021; women without an HS diploma had 27.9 per 100 000 population. This difference increased for those with an HS diploma: men with an HS diploma experienced 69.7 deaths per 100 000 population, compared with 31.1 per 100 000 population for women. This disparity then declined at higher educational levels. For those with a bachelor’s degree, men had 9.5 deaths per 100 000 population, while women had 6.4 per 100 000 population.

## Discussion

This study found large overdose death disparities based on educational attainment from 2000 to 2021 that grew especially fast during the COVID-19 pandemic. From 2019 to 2021, the overdose death rate for those with no college increased by 30.6 per 100 000 population while the overdose death rate for the group with at least some college increased by 4.5 per 100 000 population during the same time period.

From 2018 to 2021, the increase in overdose death rates was substantial among all education groups but decreased monotonically with more education. This growth was primarily attributable to synthetic opioids, likely fentanyl.^56^ In 2021, individuals without an HS diploma and those with an HS diploma but no college experience had substantially higher overdose death rates than the rest of the population, at approximately 9 times the rate among those with bachelor’s degrees. While the overdose death rates were similar for those without an HS diploma and those with an HS diploma but no college experience, there were meaningful differences when rates were compared across every other educational level.

This study found large racial and ethnic disparities even within educational categories. The American Indian and Alaska Native population had substantially larger overdose death rates than the rest of the population for every educational group, suggesting that race and ethnicity were independently associated with overdose death rate growth. Compared with White individuals, Black individuals experienced especially high overdose death rates in the population with an HS diploma. For those without an HS diploma, White individuals had higher overdose death rates, although Black individuals experienced faster growth. Overdose death rates declined at higher educational levels among almost all racial and ethnic groups, and the differences across educational groups were large within racial and ethnic groups. For White individuals, those without an HS diploma had overdose deaths rates in 2021 that were more than 12.5 times as large as those among individuals with a bachelor’s degree.

Overall, the analysis suggests that the opioid crisis has increasingly become a crisis disproportionately impacting those without any college education. Research is needed to understand the driving forces behind this gradient, such as isolating the independent roles of differences in income, employment, family composition, health care access, and other factors. Overdose death rates grew during the COVID-19 pandemic,^57,58^ and the education gradient increased further, although it is unclear what role the pandemic had relative to changes in fentanyl penetration in illicit drug markets and other factors. Overall, the education gradient merits further attention as the opioid crisis continues to intensify while exacerbating long-standing life-expectancy differentials.^59^ The important role of socioeconomic status suggests additional targeting of resources to economically disadvantaged individuals and communities, such as expanding treatment access in lower-income communities and subsidizing naloxone for individuals with low income.^60^

### Limitations

This study has some limitations. First, it relied on the accurate coding of overdose deaths and the substances involved, although the literature has expressed concerns about incomplete reporting.^51,61^ These concerns are typically about classification of specific substances, motivating the focus on broader overdose death classifications.^62^ Second, the study relied on demographic information in the NVSS. However, cause of death can impact classification of race,^63^ and misclassification of American Indian or Alaska Native decedents typically leads to underestimates for this population.^64^ Similarly, while the NVSS and ACS provide demographic information that is straightforward to categorize in the same manner, the categories are not comparable if race or educational level is miscategorized in death certificate data but not when self-reported by respondents in the ACS.^30^ Importantly, race and ethnicity population sizes in the ACS shifted in 2020.^55^ These changes appear to have substantially increased the reported multiracial population, given a relatively low baseline prior to 2020. For this reason, I considered the overdose death rates for multiracial individuals suspect. These population size shifts were relatively small for Black and White individuals given the larger baseline values, suggesting that the reported death rates for those groups were less affected. The reported size of the American Indian and Alaska Native population decreased in 2020, but this decline likely was not the primary reason for the rapid growth in overdose death rates.

Moreover, there is evidence that individuals without an HS diploma are coded in death records as having completed 4 years of HS,^65,66^ although this concern is more relevant to the earlier 1989 revision of the death certificate, not the 2003 revision, which reports diploma status. Related, the 2020 weights provided in the ACS, which were used to generate population sizes, are considered experimental.^67^ The 2021 weights are not. The 2021 values were generally supportive of trends observed in 2020.

Third, educational attainment was missing for 3.2% of overdose deaths from 2018 to 2021 and 4.0% from 2000 to 2021. I excluded these deaths instead of imputing educational levels, which would exacerbate the aforementioned disparities. Fourth, it has been argued that the relative rank in the education distribution may be more important than actual educational attainment.^23,25,27^ This issue was less relevant given the shorter period studied here.

Fifth, I discussed synthetic opioid deaths as primarily involving illicitly manufactured fentanyl. I did not observe fentanyl in the data specifically, and synthetic opioid deaths include deaths involving prescription synthetic opioids, such as tramadol. Sixth, the analysis did not explain the causes behind any of the reported educational, racial and ethnic, or sex disparities. Instead, it highlighted their relative importance.

## Conclusions

In this cross-sectional study of data from 2000 to 2021, overdose deaths increased among individuals without any college education. The increase in overdose death rate was particularly substantial between 2019 and 2021 for those without a HS diploma, primarily due to increases in deaths with synthetic opioid involvement. These findings suggest that low educational attainment has become an increasingly important factor associated with overdose deaths, escalating further as the opioid crisis transitions to fentanyl and polysubstance use^37,68^ and during the COVID-19 pandemic. Prior work on overdose rate disparities by educational level often compared individuals with and without a college degree before these evolving crises.^16,17,18,19^ This study found large and growing educational disparities when using more granular categorizations for all racial and ethnic groups, for sex, and for more recent years. The substantial increase in overdose death rates during the pandemic among individuals without any college education suggests that the opioid crisis has disproportionately impacted this population and that socioeconomic status has a large and growing role as the opioid crisis continues to evolve. The growing importance of socioeconomic status heightens the need for social policy as health policy in addressing rising overdose death rates.^69^

## Notes

Supplement 1. **eFigure 1.** Trends in Overdose Deaths Involving Opioids per 100 000 Population by Educational Attainment **eFigure 2.** Trends in Overdose Deaths per 100 000 Population by Educational Attainment, Age-Adjusted **eFigure 3.** Overdose Deaths per 100 000 Population by Education and Race and Ethnicity in 2018 and 2021

Supplement 2.
Data Sharing Statement
