# Supplementary material for: Educational Attainment and US Drug Overdose Deaths
Source: JAMA Health Forum. 2023 Oct 6;4(10):e233274. doi: 10.1001/jamahealthforum.2023.3274 (PMC10559184; doi:10.1001/jamahealthforum.2023.3274)
Supplement: Supplement 1. — eFigure 1. Trends in Overdose Deaths Involving Opioids per 100 000 Population by Educational Attainment eFigure 2. Trends in Overdose Deaths per 100 000 Population by Educational Attainment, Age-Adjusted eFigure 3. Overdose Deaths per 100 000 Population by Education and Race and Ethnicity in 2018 and 2021 [file jamahealthforum-e233274-s001.pdf]

Supplementary Online Content

Powell D. Educational attainment and US drug overdose deaths. *JAMA Health Forum*. 2023;4(10):e233274. doi:10.1001/jamahealthforum.2023.3274

eFigure 1. Trends in Overdose Deaths Involving Opioids per 100 000 Population by Educational Attainment

eFigure 2. Trends in Overdose Deaths per 100 000 Population by Educational Attainment, Age-Adjusted

eFigure 3. Overdose Deaths per 100 000 Population by Education and Race and Ethnicity in 2018 and 2021

This supplementary material has been provided by the authors to give readers additional information about their work.

© 2023 Powell DM. *JAMA Health Forum*.

eFigure 1

Caption: Trends in overdose deaths involving opioids per 100,000 by education attainment

Panel A: 2000-2021, 2 education categories

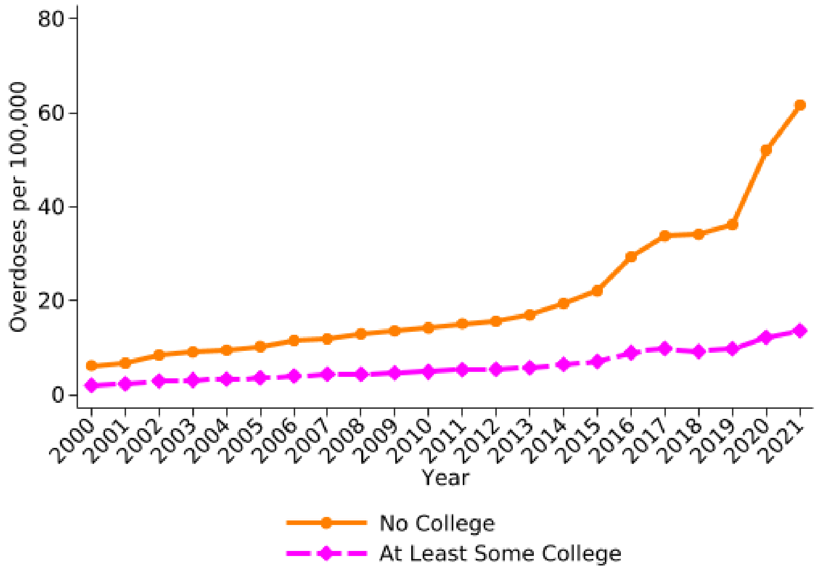

Panel B: 2018-2021, 4 education categories

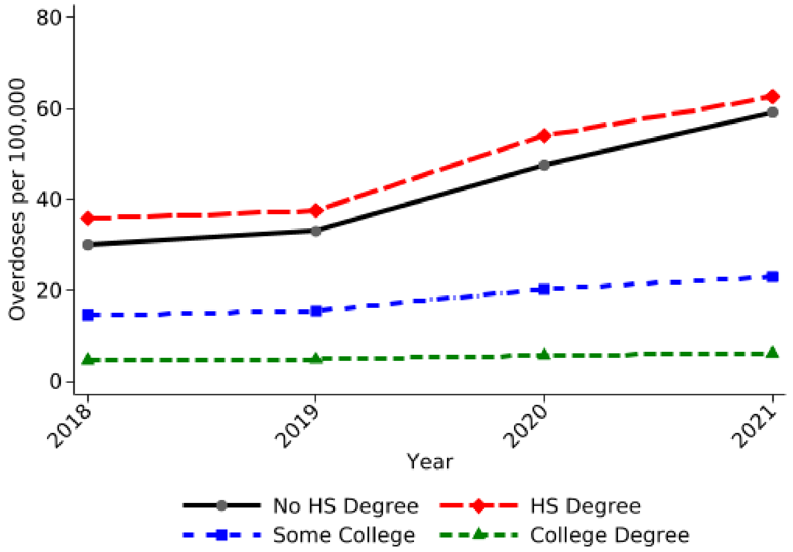

Source: Author’s analysis of NVSS data.

Notes: Overdose deaths and opioid involvement are defined in the Methods section. Education attainment stratification uses information reported on death certificates.

© 2023 Powell DM. *JAMA Health Forum*.

eFigure 2

Caption: Trends in overdose deaths per 100,000 by education attainment, age-adjusted

<u>Panel A: 2000-2021, 2 education categories</u>

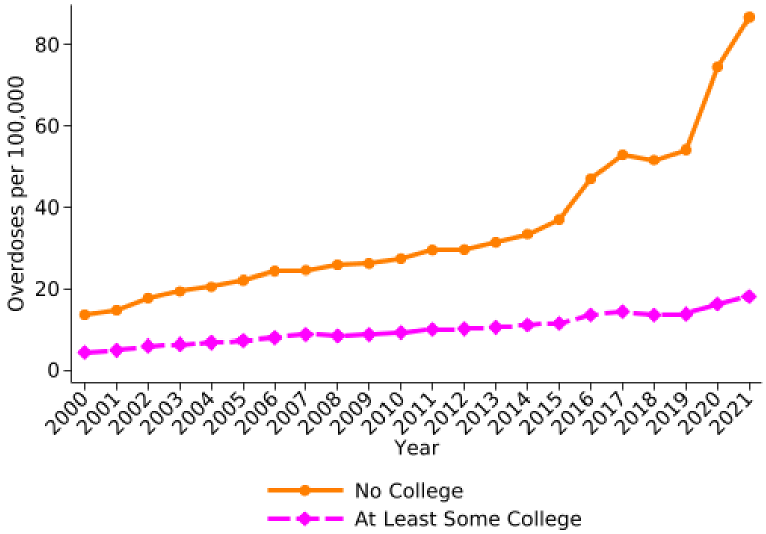

<u>Panel B: 2018-2021, 4 education categories</u>

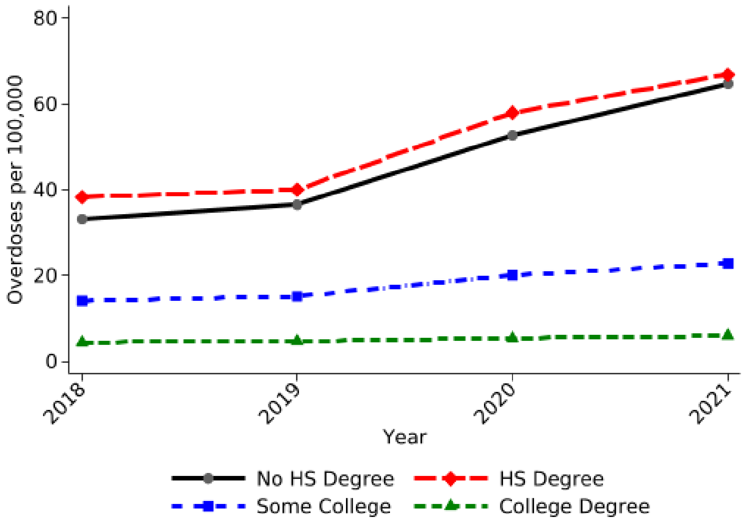

Source: Author's analysis of NVSS data.

Notes: Overdose deaths and opioid involvement are defined in the Methods section. Education attainment stratification used information reported on death certificates. All rates were age-adjusted by aggregating age group-specific rates using common scaling factors across all education categories. The age groups were 25-34, 35-44, 45-54, 55-64, 65-74, and 75+.

© 2023 Powell DM. *JAMA Health Forum*.

eFigure 3: Overdose deaths per 100,000 by education and race/ethnicity in 2018 and 2021

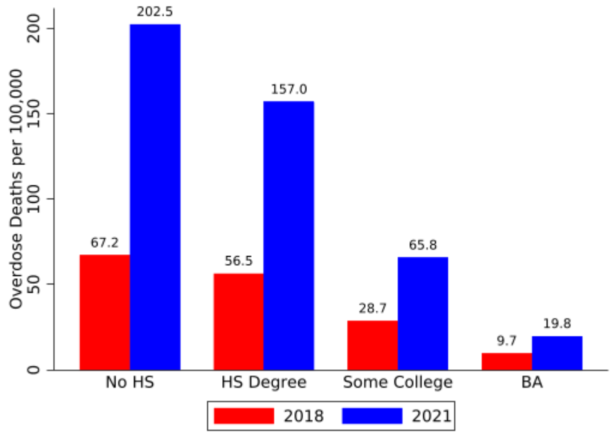

A. American Indian and Alaska Natives

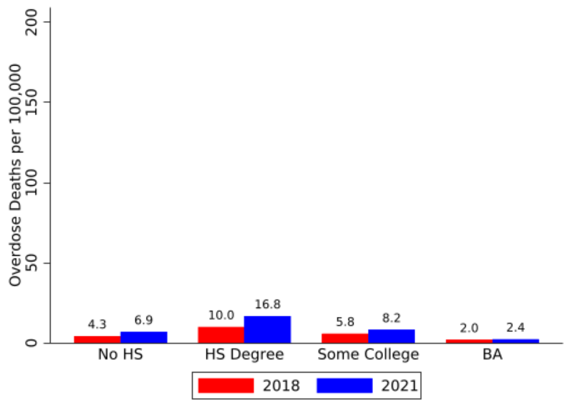

B. Asian American and Pacific Islanders

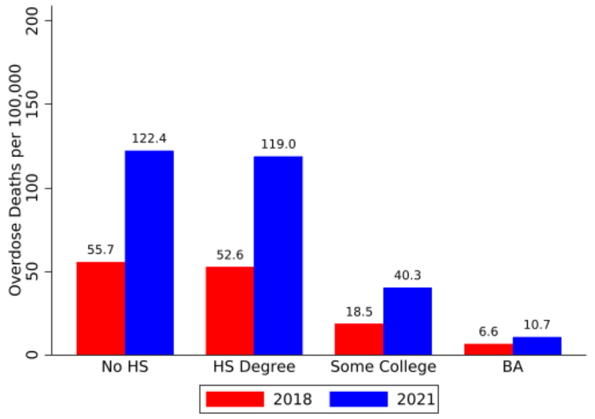

C. Black

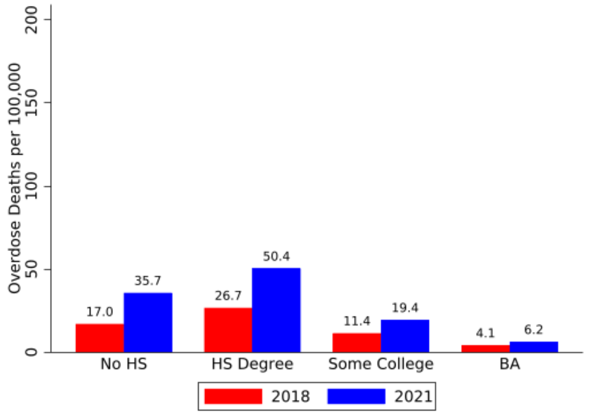

D. Hispanic

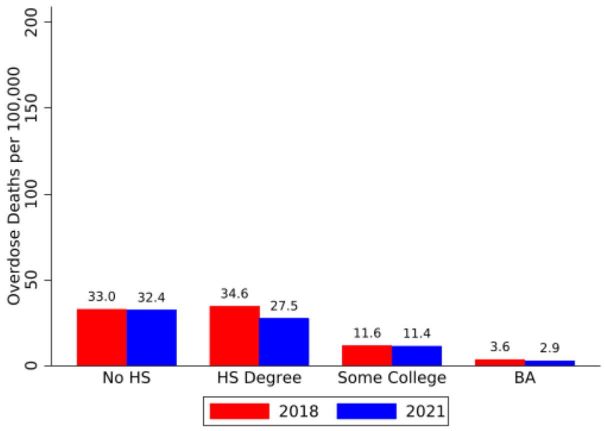

E. Multiple Races

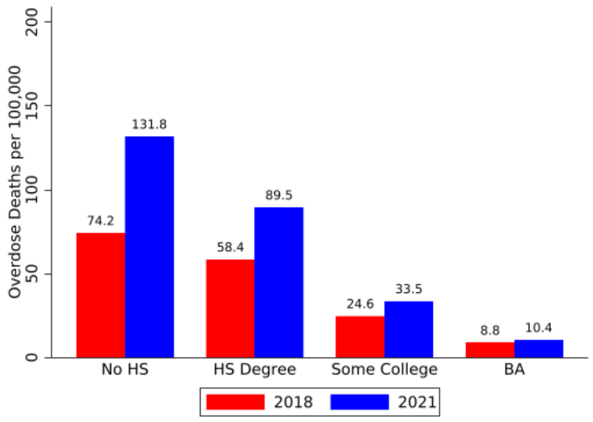

F. White

Notes: See notes for Figure 3 in the main text.

© 2023 Powell DM. *JAMA Health Forum*.
