# Supplementary material for: Educational Attainment and US Drug Overdose Deaths
Source: JAMA Health Forum. 2023 Oct 6;4(10):e233274. doi: 10.1001/jamahealthforum.2023.3274 (PMC10559184; doi:10.1001/jamahealthforum.2023.3274)
Supplement: Supplement 2. — Data Sharing Statement [file jamahealthforum-e233274-s002.pdf]

# Data Sharing Statement

Powell. Educational Attainment and US Drug Overdose Deaths. *JAMA Health Forum*.
Published October 06, 2023. doi:10.1001/jamahealthforum.2023.3274

**Data**
**Data available:** Yes
**Data types:** Data (not involving human participants)
**How to access data:** Data will be deposited in the Harvard Dataverse.
**When available:** With publication

**Supporting Documents**
**Document types:** Statistical/analytic code
**How to access documents:** Code will be deposited in the Harvard Dataverse.
**When available:** With publication

**Additional Information**
**Who can access the data:** The data are public and will be available to anyone.
**Types of analyses:** For any purpose.
**Mechanisms of data availability:** The data will be made public and available to all.
**Any additional restrictions:** None.
